# Retrospective analysis of peri-implant tissue health and patient reported outcome measures in indirect sinus floor augmentation in diabetic and non-diabetic patients

**DOI:** 10.1186/s12903-025-07565-z

**Published:** 2025-12-29

**Authors:** Abhay P. Kolte, Pranjali V. Bawankar, Rajashri A. Kolte, Pavan Bajaj, Mahima Kothekar, Shivani Thakre

**Affiliations:** 1 Department of Periodontics and Implantology, Ranjeet Deshmukh Dental College and Research Centre, Nagpur, India; 2 Department of Periodontics and Implantology, Sharad Pawar Dental College and Hospital, Datta Meghe Institute of Higher Education and Research (DU), Wardha, Sawangi (Meghe) India

**Keywords:** Bone graft, Maxillary sinus, PROMs, Sinus floor elevation, Type 2 diabetes mellitus

## Abstract

**Background:**

Implant therapy is a predictable solution for replacing missing teeth; however, the posterior maxilla presents challenges due to limited residual bone height and porous bone quality. The primary outcome of this retrospective study was to compare peri-implant tissue health—specifically total bone height (TBH) and marginal bone loss (MBL)—between well-controlled type 2 diabetes mellitus (T2DM) and non-diabetic patients over a 10-year period. Patient-reported outcome measures (PROMs) were evaluated as a secondary outcome.

**Methods:**

Seventy-six patients received 115 implants placed in the posterior maxilla with indirect sinus floor elevation. Based on metabolic status, patients were categorized into well-controlled T2DM (*n* = 31) and non-diabetic groups (*n* = 45). Clinical and radiographic parameters—gingival index (GI), residual bone height (RBH), sinus membrane elevation (SME), total bone height (TBH), marginal bone loss (MBL), sinus membrane thickness (SMT), and peri-implant soft tissue dehiscence (PSTD)—were assessed at baseline to 10 years follow up. PROMs were assessed using the OHIP-14 scale.

**Results:**

At 10-year follow-up, the mean total bone height was 14.05 ± 1.03 mm in the T2DM group and 14.65 ± 0.99 mm in the non-T2DM group, reflecting a mean difference of 0.60 mm (*P* = 0.002). Mean marginal bone loss was 1.21 ± 0.72 mm in T2DM patients vs. 0.56 ± 0.61 mm in non-diabetic patients, with a mean difference of 0.65 mm (*P* < 0.001). PROMs indicated similar patient satisfaction between groups, although esthetic and functional scores declined more in T2DM patients over time.

**Conclusion:**

Both groups showed comparable long-term clinical outcomes, though lesser total bone height and greater mean marginal bone loss was observed in diabetic patients. Indirect sinus floor elevation with composite grafting demonstrated predictable success in both well-controlled T2DM and non-T2DM patients.

**Clinical trial number:**

Not applicable.

## Background

 Over the years, implant therapy has proven to be a reliable and predictable way to replace missing teeth [1–4]. The validation of dental implant success depends on long-term survival along with its functional, esthetic, hard and soft-tissue stability which further reflects in the patient reported outcome measures. However, the posterior maxilla because of limited residual bone height associated with porous bone quality presents several constraints for dental implant placement. Insufficient residual bone height is often ascribed to crestal bone resorption and increased sinus pneumatization subsequent to tooth loss. Maxillary sinus floor elevation through trans-crestal approach and direct lateral access are two common and proven options for vertical bone augmentation with implant placements in the atrophic posterior maxilla [5, 6]. The literature reveals several advantages of the osteotome mediated sinus floor elevation over the others in terms of sinus augmentation in localized area thereby making the procedure more conservative with a reduced morbidity, shorter time period for implant loading, negligible chances of wound dehiscence, and over 90% survival rates [7, 8]. The protocol adopted for grafting is critical in terms of the material used as it may alter the cost of treatment, operating time, loading practices, as well as selection of the prostheses [9].

Favourable clinical outcomes were achieved with the use of grafting materials such as deproteinized bovine bone mineral with L-PRF in sinus floor elevation due to its osteoconductive properties [10] .

An average 3-year survival rate in 92.8% of the cases was recorded through osteotome mediated sinus floor elevation procedures in one of the systematic reviews; but because of the presence of immense heterogeneity, no inference could be drawn about the augmentation materials [11]. In another systematic review, the mean cumulative survival rates were observed with osteotome mediated sinus floor elevation with and without augmentation and the authors inferred that using the augmentation material did not ensure a long-term survival of the implants. The authors were unable to perform meta-analysis as the included studies had low levels of evidence being case series [12]. While diabetes has been recognised as an important risk factor for periodontitis [13], its correlation with peri-implant diseases has not been fully investigated. In one of the retrospective studies, the authors reported no association between well controlled diabetes and peri-implantitis or implant failure [14].

It is thus assumed that there is a paucity in the existing literature clarifying the influence of Diabetes and use of composite graft materials and its long-term evaluation in indirect sinus floor elevation with bone augmentation. So, the purpose of this retrospective study was to assess the peri-implant tissue health and patient reported outcome measures (PROMs) in indirect sinus floor elevation procedures with augmentation in diabetic and non-diabetic patients clinically and radiographically.

## Methods

The present retrospective study was performed taking in to account previous records of 76 patients with 39 males and 37 females treated with 115 dental implants placed in posterior maxilla using the sinus floor elevation with bone augmentation from 2012 to 2015 in the Department of Periodontology and Implantology of Ranjeet Deshmukh Dental College and Research Centre, Nagpur, India. Out of the total number of patients 5 patients with equal number of implants were lost to follow up after four years, while 71 patients with 110 implants completed the ten years follow up. Written informed consent was procured from all the participants as a matter of preoperative requirements mandated by the institute for the purpose of scientific advancement. For publishing purposes, this retrospective study was approved by our institutes Ethics Committee (Approval No.IEC/RDDC&RC/Staff/Perio/177/2025) and was in compliance with Helsinki Declaration. The study protocol was conducted ensuring the Strengthening the Reporting of Observational Studies in Epidemiology (STROBE) statements.

Recruitment of the participants followed certain inclusion criteria such as (a) Patients with age > 18 years demonstrating good oral hygiene, (b) partial edentulism in the maxillary posterior area for at least 3 months from tooth loss, (c) available residual bone height ranging from 4 to 8 mm, (d) without rhinitis or sinusitis, (e) without any previous history of sinus surgery or grafting at the implant site and (f) patients whose complete case records and imaging data were present in the records section. Exclusion criteria implemented for the study were (a) uncontrolled periodontal lesions or other oral disorders, (b) systemic illness other than well controlled diabetes, (c) patients with a history of head and neck radiation treatment, (d) heavy smokers (≥ 20 cigarettes per day) and (e) sinus membrane perforation during the procedure (f) patients with any history of sinus-related pathology—including chronic sinusitis, rhinitis, previous sinus surgery, or radiographic evidence of sinus disease.

Diagnosis of type 2 diabetes mellitus was confirmed based on American Diabetes Association criteria. Patients were classified as “well-controlled T2DM” only if HbA1c records indicated values < 7% for a minimum of six months prior to implant placement and remained within target levels during follow-up [15].

Group I comprised of well controlled type 2 diabetes mellitus (T2DM) while Group II included patients without T2DM. (non-T2DM)

**Fig. 1:**
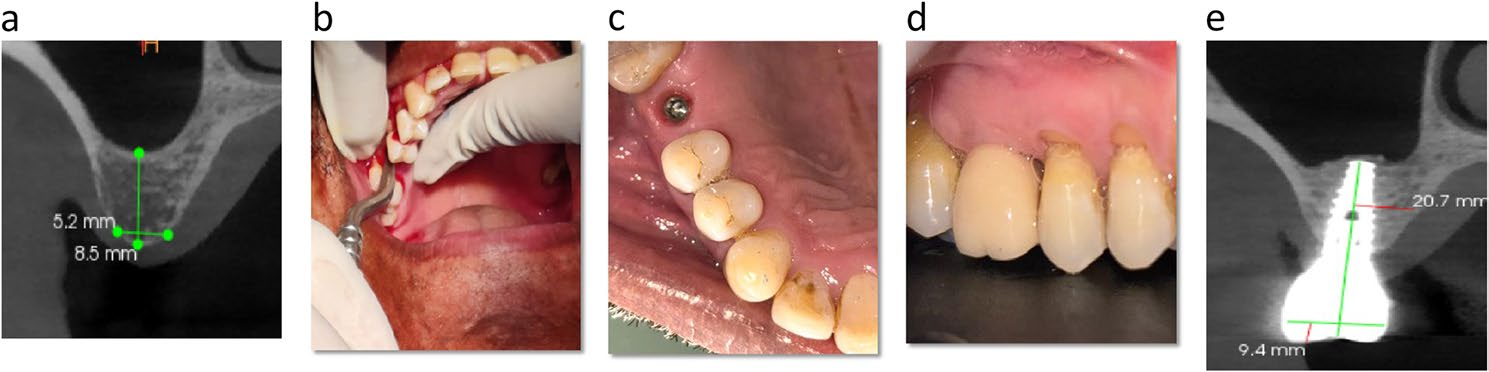
**a**: Preoperative CBCT Showing Residual Bone Height. This image demonstrates the baseline anatomical condition of the posterior maxilla prior to sinus floor elevation. The residual bone height (RBH) was measured from the alveolar crest to the sinus floor, establishing the available bone dimensions needed for treatment planning. The limited RBH highlights the clinical challenge that necessitates sinus augmentation. **b** : Indirect (Transcrestal) Sinus Lift Using Osteotome Technique. This intraoperative photograph illustrates the elevation of the Schneiderian membrane using calibrated osteotomes through the crestal approach. Controlled tapping induces a green-stick fracture of the sinus floor, allowing vertical bone augmentation without creating a lateral window. This minimally invasive method reduces morbidity and is suitable when RBH is sufficient to achieve primary implant stability. **c** : Implant Placement Following Sinus Membrane Elevation and Grafting. After achieving adequate membrane elevation, a composite graft comprising DFDBA, hydroxyapatite, β-TCP, and PRF wass inserted into the osteotomy. The implant was then placed 1 mm subcrestally to allow optimal bone-to-implant contact. This figure highlights proper implant positioning and the three-dimensional stability required for successful osseointegration. **d** : Final Prosthesis Placement After Healing Phase. This figure presents the implant-supported prosthesis after completion of the healing and osseointegration period. Proper emergence profile, occlusal relationships, and soft-tissue adaptation signify successful functional rehabilitation of the edentulous posterior maxilla. **e** : Ten-Year Follow-up CBCT Displaying Long-Term Bone Stability Around the Implant. The CBCT scan taken at 10 years demonstrated stable peri-implant bone levels and maintained sinus augmentation volume

A pre-surgical cone beam computed tomography (CBCT) was obtained for the appraisal of residual bone height and other adjacent anatomical structures of the maxillary region. (Fig. 1 (a)) All the surgical procedures of sinus floor elevation with bone augmentation were performed by experienced clinician under appropriate local anaesthesia and strictly sterile conditions. Subsequent to the mid-crestal incision, a full-thickness mucoperiosteal flap was raised to visualize the alveolar crest. Then the implant socket was prepared with pilot drill up to a depth of 1 mm inferior to the sinus floor, as measured radiographically which was then expanded in diameter through the sequential osteotomes. A fracture was induced in the thin inferior bone wall margin of the sinus and the maxillary sinus floor was elevated using osteotomes. The integrity of the Schneiderian membrane was examined utilizing the Valsalva manoeuvre and on conformity of it, a composite graft was prepared which was a combination of demineralised freeze-dried bone allograft (DFDBA) mixed with hydroxyapatite and β-tricalcium phosphate and PRF. This graft material was placed in the osteotomy and gently pushed with apically with the osteotomes (Fig. 1(b, c)). Once the desired amount of graft material was inserted the appropriately sized implant was placed a millimetre sub-crestally and the flaps were sutured. Standardized radio-visuography (RVG) image was taken at this time point to determine the gain in vertical bone height and implant positions. The patients were given instructions and prescribed with nasal decongestants, antibiotics, and analgesics for five days. The delayed loading protocol was used all the implants included in the study. (Fig. 1(d))

All the implants were placed in partially edentulous maxillary posterior sites in a two-stage approach with exclusion of implants less than 10 mm in length. Implants with a minimum diameter of 3.5 mm were included in the assessment so as to minimize variations that could have a bearing on the interpretations. A protocol of regular annual follow-up for patients enabled assessment of the implant and tissues. All patients were informed of the need of regular follow-up for success of the therapy and after oral prophylaxis instructions were reinforced on every recall visit. (Fig. 1(e)) Each patient was assigned an identification number, which enabled blinding of all the examiners.

Clinical and radiographic parameters evaluated were Gingival index (GI) [16], Residual bone height (RBH), Sinus membrane elevation (SME), Total bone height (TBH), Marginal bone loss (MBL), Sinus membrane thickness (SMT) and Peri-implant soft tissue dehiscence (PSTD) at baseline and annually for a period of ten years. The hard tissue examinations were performed on RVG annually and CBCT every second year after baseline. Measurement evaluations were done as the distance from; RBH: alveolar crest to the sinus floor at the centre of the edentulous site on preoperative CBCT, SME: initial sinus floor and elevated sinus floor apical to the implant apex, TBH: sum of RBH plus SME [17], MBL: implant shoulder margin to alveolar crest, SMT: inferior and superior border of sinus membrane at the implant apex and PSTD: presence of apical shift in the margin of peri-implant mucosa or only a grayish hue identified through the mucosa and/ or discrepancies in the implant-supported crown length as compared with the adjacent natural tooth).

The patient satisfaction assessment was done as per OHIP 14 scale [18] considering parameters such as functional outcome, pain and discomfort, esthetic appearance, and overall satisfaction, with a pre-validated questionnaire. A structured maintenance protocol was implemented for all patients, consisting of professional oral prophylaxis, reinforcement of oral hygiene instructions, and clinical assessment of peri-implant parameters at each recall visit. Patients were scheduled for annual maintenance visits throughout the follow-up period, in accordance with the evidence-based supportive care recommendations outlined by Blasi et al. [19]. At each visit, peri-implant soft-tissue indices, probing depths, and radiographs (RVG annually and CBCT every second year) were obtained following a standardized protocol to ensure consistency in monitoring peri-implant health.

In addition to the longitudinal assessment of hard and soft tissue parameters, implant survival rates and biological/technical complications were documented throughout the 10-year follow-up period. Implant survival was defined as the absence of mobility, persistent pain, suppuration, peri-implant radiolucency, or loss of functional integration. At each annual recall, biological complications—including peri-implant mucositis, increased probing depths, bleeding on probing, sinus membrane–related changes, and peri-implant soft tissue dehiscence—were recorded. Technical complications such as screw loosening, prosthesis fracture, or occlusal wear were also evaluated.

All radiographic measurements (RVG and CBCT) were performed by two experienced examiners who were calibrated prior to data collection. To minimize observer bias, an intra- and inter-examiner reliability assessment was conducted using intraclass correlation coefficients (ICCs) on 20% of randomly selected radiographs. Both intra- and inter-examiner ICC values were > 0.85, indicating excellent measurement consistency. In cases of discrepancy, consensus was reached through joint evaluation.

The continuous parameters were expressed in terms of mean, median, standard deviation, minimum and maximum, while categorical were summarized in terms of frequency and percentage. The comparison of mean levels of continuous parameters between T2DM and non-diabetic groups was performed using t-test for independent samples. Further, the comparison of mean levels of parameters across time in each group was performed using repeated measure analysis of variance. The comparison of PROM parameters at each time point between two groups was done using Mann-Whitney U test, while the comparison of each parameter between 1 year and 10 years was done using Wilcoxon signed-rank test. All the analyses were performed using SPSS version 26.0 (IBM Corp. ARMONK USA). To account for potential confounding factors, a multivariate analysis was additionally performed. Multiple linear regression models were constructed with marginal bone loss (MBL) and total bone height (TBH) at 10 years as dependent variables. Independent predictors included diabetic status (T2DM vs. non-T2DM), oral hygiene, prosthesis type, implant diameter, implant length, baseline residual bone height (RBH), and smoking status. Statistical significance was set at *p* < 0.05.

## Results

The study involved 76 patients undergoing implant with a mean age of 46.78 ± 11.96 years and median of 48 years. There were 39 (51.31%) males while 37 (48.68%) females in the study sample. Regarding diabetic status, 31 (40.78%) patients had T2DM, while 45 (59.21%) were non-T2DM (Table 1).

**Table 1:** Descriptive statistics for demographic characteristics of patients included in study

Characteristic		Statistic
Age (years)		46.78 ± 11.96; 48; (21, 71)
Sex	Male	39 (51.31%)
	Female	37 (48.68%)
Type 2 DM		
	Present	31 (40.78%)
	Absent	45 (59.21%)

Table 2 provides the comparison of parameters TBH, MBL, SMT, GI and PSTD between two groups at different time points. At baseline and six months, all the parameters showed non-significant difference between two groups. After one year, the MBL in T2DM group (0.61 ± 0.45 mm) was significantly higher than that of non-T2DM group (0.13 ± 0.32 mm) with a *p* < 0.001. The mean SMT in the T2DM group (1.36 ± 0.34 mm) was significantly higher than the non-T2DM group (1.22 ± 0.26) with a p-value of 0.013.

**Table 2:** Comparison of various parameters between DM groups according to time

Time	Parameters	Type 2 DM				*P*-value
		Present		Absent		
		*n*	Mean ± SD	*n*	Mean ± SD	
Baseline	Bone height	51	7.26 ± 1.02	64	7.32 ± 1.06	0.735
	Sinus membrane elevation	51	7.32 ± 0.94	64	7.51 ± 0.95	0.301
	Total bone height	51	14.58 ± 1.07	64	14.83 ± 1.08	0.217
	Marginal bone loss	51	0.00 ± 0.00	64	0.00 ± 0.00	-
	Sinus membrane thickness	51	1.21 ± 0.31	64	1.15 ± 0.28	0.292
	Gingival index	51	0.00 ± 0.00	64	0.00 ± 0.00	-
	PSTD	51	0.00 ± 0.00	64	0.00 ± 0.00	-
6 months	Bone height	51	7.26 ± 1.02	64	7.32 ± 1.06	0.735
	Sinus membrane elevation	51	7.32 ± 0.94	64	7.51 ± 0.95	0.301
	Total bone height	51	14.58 ± 1.07	64	14.83 ± 1.08	0.217
	Marginal bone loss	51	0.00 ± 0.00	64	0.00 ± 0.00	-
	Sinus membrane thickness	51	1.27 ± 0.28	64	1.19 ± 0.25	0.092
	Gingival index	51	0.08 ± 0.27	64	0.08 ± 0.32	0.996
	PSTD	51	0.02 ± 0.14	64	0.06 ± 0.24	0.266
1 year	Total bone height	51	14.39 ± 1.10	64	14.79 ± 1.12	0.060
	Marginal bone loss	51	0.61 ± 0.45	64	0.13 ± 0.32	**< 0.001**
	Sinus membrane thickness	51	1.36 ± 0.34	64	1.22 ± 0.26	**0.013**
	Gingival index	51	0.31 ± 0.47	64	0.16 ± 0.41	0.056
	PSTD	51	0.24 ± 0.43	64	0.11 ± 0.31	0.072
2 years	Total bone height	51	14.37 ± 1.10	64	14.79 ± 1.14	**0.048**
	Marginal bone loss	51	0.60 ± 0.47	64	0.29 ± 0.45	**< 0.001**
	Sinus membrane thickness	51	1.41 ± 0.35	64	1.25 ± 0.29	**0.009**
	Gingival index	51	0.39 ± 0.49	64	0.11 ± 0.36	**0.001**
	PSTD	51	0.27 ± 0.45	64	0.08 ± 0.27	**0.005**
4 years	Total bone height	50	14.38 ± 1.11	60	14.82 ± 1.09	**0.040**
	Marginal bone loss	50	0.64 ± 0.49	60	0.32 ± 0.47	**0.001**
	Sinus membrane thickness	50	1.49 ± 0.33	60	1.24 ± 0.28	**< 0.001**
	Gingival index	50	0.32 ± 0.47	60	0.02 ± 0.13	**< 0.001**
	PSTD	50	0.26 ± 0.44	60	0.02 ± 0.13	**< 0.001**
6 years	Total bone height	50	14.26 ± 1.06	60	14.72 ± 1.00	**0.023**
	Marginal bone loss	50	0.90 ± 0.56	60	0.42 ± 0.54	**< 0.001**
	Sinus membrane thickness	50	1.79 ± 0.31	60	1.39 ± 0.32	**< 0.001**
	Gingival index	50	0.42 ± 0.50	60	0.05 ± 0.22	**< 0.001**
	PSTD	50	0.28 ± 0.45	60	0.03 ± 0.18	**< 0.001**
8 years	Total bone height	50	14.18 ± 1.05	60	14.68 ± 0.99	**0.013**
	Marginal bone loss	50	1.03 ± 0.65	60	0.46 ± 0.57	**< 0.001**
	Sinus membrane thickness	50	1.86 ± 0.31	60	1.43 ± 0.34	**< 0.001**
	Gingival index	50	0.48 ± 0.50	60	0.05 ± 0.22	**< 0.001**
	PSTD	50	0.34 ± 0.48	60	0.03 ± 0.18	**< 0.001**
10 years	Total bone height	50	14.05 ± 1.03	60	14.65 ± 0.99	**0.002**
	Marginal bone loss	50	1.21 ± 0.72	60	0.56 ± 0.61	**< 0.001**
	Sinus membrane thickness	50	1.93 ± 0.35	60	1.46 ± 0.36	**< 0.001**
	Gingival index	50	0.50 ± 0.51	60	0.05 ± 0.22	**< 0.001**
	PSTD	50	0.44 ± 0.50	60	0.05 ± 0.22	**< 0.001**

At the end of ten years, the mean TBH in the T2DM group (14.05 ± 1.03 mm) was significantly smaller than that of non-T2DM group (14.65 ± 0.99 mm) with a p-value 0.002. The MBL in the T2DM group (1.21 ± 0.72) was significantly higher than the non-T2DM group (0.56 ± 0.61) with a p-value < 0.001. The mean SMT in the T2DM group (1.93 ± 0.35 mm) was significantly higher than the non-T2DM group (1.46 ± 0.36 mm) with a p-value < 0.001. The mean GI in the T2DM group (0.50 ± 0.51) was significantly higher than the non-T2DM group (0.05 ± 0.22) with a p-value < 0.001. Moreover, the mean PSTD in T2DM group (0.44 ± 0.48) was significantly higher than the non-T2DM group (0.05 ± 0.22) with a p-value < 0.001.

Table 3 provides the comparison of various parameters across time in each study group. The TBH in T2DM group showed statistically significant change in the mean levels from baseline to 10 years (*p* < 0.001). Later years also indicated significant reduction in the TBH. In the non-T2DM group also, the change in TBH was significant from baseline to 10 years (*p* < 0.001). At 10 years, the mean TBH in the non-T2DM group was significantly higher than the T2DM group.

**Table 3:** Comparison of various parameters across time within each group

Parameter	Time	Type 2 DM			
		Present		Absent	
		*n*	Mean ± SD	*n*	Mean ± SD
Total bone height	Baseline	51	14.58 ± 1.07	64	14.83 ± 1.08
	6 months	51	14.58 ± 1.07	64	14.83 ± 1.08
	1 year	51	14.39 ± 1.10	64	14.79 ± 1.12
	2 years	51	14.37 ± 1.10	64	14.79 ± 1.14
	4 years	50	14.38 ± 1.11	60	14.82 ± 1.09
	6 years	50	14.26 ± 1.06	60	14.72 ± 1.00
	8 years	50	14.18 ± 1.05	60	14.68 ± 0.99
	10 years	50	14.05 ± 1.03	60	14.65 ± 0.99
	*P*-value^***1***^	**< 0.001**		**< 0.001**	
Marginal bone loss	Baseline	51	0.00 ± 0.00	64	0.00 ± 0.00
	6 months	51	0.00 ± 0.00	64	0.00 ± 0.00
	1 year	51	0.61 ± 0.45	64	0.13 ± 0.32
	2 years	51	0.60 ± 0.47	64	0.29 ± 0.45
	4 years	50	0.64 ± 0.49	60	0.32 ± 0.47
	6 years	50	0.90 ± 0.56	60	0.42 ± 0.54
	8 years	50	1.03 ± 0.65	60	0.46 ± 0.57
	10 years	50	1.21 ± 0.72	60	0.56 ± 0.61
	*P*-value^***1***^	**< 0.001**		**< 0.001**	
Sinus membrane thickness	Baseline	51	1.21 ± 0.31	64	1.15 ± 0.28
	6 months	51	1.27 ± 0.28	64	1.19 ± 0.25
	1 year	51	1.36 ± 0.34	64	1.22 ± 0.26
	2 years	51	1.41 ± 0.35	64	1.25 ± 0.29
	4 years	50	1.49 ± 0.33	60	1.24 ± 0.28
	6 years	50	1.79 ± 0.31	60	1.39 ± 0.32
	8 years	50	1.86 ± 0.31	60	1.43 ± 0.34
	10 years	50	1.93 ± 0.35	60	1.46 ± 0.36
	*P*-value^***1***^	**< 0.001**		**< 0.001**	
Gingival index	Baseline	51	0.00 ± 0.00	64	0.00 ± 0.00
	6 months	51	0.08 ± 0.27	64	0.08 ± 0.32
	1 year	51	0.31 ± 0.47	64	0.16 ± 0.41
	2 years	51	0.39 ± 0.49	64	0.11 ± 0.36
	4 years	50	0.32 ± 0.47	60	0.02 ± 0.13
	6 years	50	0.42 ± 0.50	60	0.05 ± 0.22
	8 years	50	0.48 ± 0.50	60	0.05 ± 0.22
	10 years	50	0.50 ± 0.51	60	0.05 ± 0.22
	*P*-value^***1***^	**< 0.001**		0.093	
PSTD	Baseline	51	0.00 ± 0.00	64	0.00 ± 0.00
	6 months	51	0.02 ± 0.14	64	0.06 ± 0.24
	1 year	51	0.24 ± 0.43	64	0.11 ± 0.31
	2 years	51	0.27 ± 0.45	64	0.08 ± 0.27
	4 years	50	0.26 ± 0.44	60	0.02 ± 0.13
	6 years	50	0.28 ± 0.45	60	0.03 ± 0.18
	8 years	50	0.34 ± 0.48	60	0.03 ± 0.18
	10 years	50	0.44 ± 0.50	60	0.05 ± 0.22
	*P*-value^***1***^	**< 0.001**		0.252	

Regarding MBL, in the T2DM group, the overall change from baseline to 10 years was statistically significant (*p* < 0.001). In the non-T2DM group also, the overall change in the mean levels from baseline to 10 years was statistically significant (*p* < 0.001). The overall bone loss in the non-T2DM group was significantly smaller than the T2DM group at the end of 10 years.

The mean SMT in the T2DM group showed statistically significant change from baseline to 10 years (*p* < 0.001). The pair wise comparison revealed that at follow up times, the mean level was significantly higher as compared to previous time points. In the non-T2DM group also, the mean changed significantly from baseline to 10 years (*p* < 0.001). Beyond 4 years, there was significant increase in the mean levels at an interval of two years.

The mean GI showed significant increase from baseline to 10 years in the T2DM group (*p* < 0.001). In the non-T2DM group, the change of mean GI was not significant throughout the study period.

The mean PSTD levels showed statistically significant change in the T2DM group. The paired comparisons revealed that the change from baseline to six months was significant. In the non-T2DM group, no significant change in the mean levels of PSTD was observed during the study period.

At 1-year and 10-years, a significant difference of mean functional total score was observed between T2DM and non-T2DM groups. The other parameters showed statistically non-significant difference between the two groups. (Table 4) In the T2DM group, the change in esthetic total 1 to 10 years showed statistically significant reduction (*p* = 0.002), while other parameters showed non-significant difference between the two time points. In the non-T2DM group, the change in the OHIP-14 total from 1 year to 10 years was significant (*p* = 0.02). Further, the difference of esthetic-total between 1 and 10 years was also statistically significant (*p* = 0.005). The change in pain-total was also significant from 1 year to 10 years (*p* = 0.008) (Table 5).

**Table 4:** Comparison of various patient reported outcome measure (PROM) between DM and non-DM patient groups

Time		Type 2 DM										*P*-value^1^
	Parameter	Present (*N* = 27)					Absent (*N* = 44)					
		Mean	Median	SD	Minimum	Maximum	Mean	Median	SD	Minimum	Maximum	
1 year	OHIP-Total	7.67	8.00	1.00	6	10	7.59	8.00	0.73	6	10	0.720
	Aesthetic-Total	28.41	29.00	1.19	24	30	28.68	29.00	0.83	27	30	0.393
	Functional-Total	3.63	4.00	0.63	2	4	3.89	4.00	0.32	3	4	**0.045**
	Pain-Total	0.26	0.00	0.59	0	2	0.16	0.00	0.37	0	1	0.682
	Overall satisfaction	4.89	5.00	0.32	4	5	4.86	5.00	0.35	4	5	0.758
10 years	OHIP-Total	7.56	8.00	0.85	6	9	7.43	7.00	0.70	6	9	0.464
	Aesthetic-Total	28.00	28.00	1.24	24	30	28.41	29.00	0.87	26	30	0.170
	Functional-Total	3.63	4.00	0.63	2	4	3.89	4.00	0.32	3	4	**0.045**
	Pain-Total	0.33	0.00	0.62	0	2	0.32	0.00	0.52	0	2	0.856
	Overall satisfaction	4.85	5.00	0.36	4	5	4.80	5.00	0.41	4	5	0.554

**Table 5:** Comparison of various patient reported outcome measure (PROM) between two time points in each study group

		Type 2 DM									
Parameter	Time	Present (*N* = 27)					Absent (*N* = 44)				
		Mean	Median	SD	Minimum	Maximum	Mean	Median	SD	Minimum	Maximum
OHIP-Total	1 year	7.67	8.00	1.00	6	10	7.59	8.00	0.73	6	10
	10 years	7.56	8.00	0.85	6	9	7.43	7.00	0.70	6	9
	*P*-value^*1*^	0.366					**0.020**				
Aesthetic-Total	1 year	28.41	29.00	1.19	24	30	28.68	29.00	0.83	27	30
	10 years	28.00	28.00	1.24	24	30	28.41	29.00	0.87	26	30
	*P*-value^*1*^	0.002					**0.005**				
Functional-Total	1 year	3.63	4.00	0.63	2	4	3.89	4.00	0.32	3	4
	10 years	3.63	4.00	0.63	2	4	3.89	4.00	0.32	3	4
	*P*-value^*1*^	0.999					0.999				
Pain-Total	1 year	0.26	0.00	0.59	0	2	0.16	0.00	0.37	0	1
	10 years	0.33	0.00	0.62	0	2	0.32	0.00	0.52	0	2
	*P*-value^*1*^	0.157					**0.008**				
Overall satisfaction	1 year	4.89	5.00	0.32	4	5	4.86	5.00	0.35	4	5
	10 years	4.85	5.00	0.36	4	5	4.80	5.00	0.41	4	5
	*P*-value^*1*^	0.317					0.083				

Multiple linear regression analysis demonstrated that diabetic status remained a statistically significant independent predictor of marginal bone loss at 10 years (β = 0.41, *p* < 0.001), even after adjusting for potential confounders including baseline gingival index, prosthesis type, implant dimensions, smoking status, and baseline RBH. None of the other variables showed a statistically significant association with MBL (*p* > 0.05). For total bone height (TBH), diabetic status also exhibited a modest but significant negative association at 10 years (β = −0.22, *p* = 0.018). Baseline RBH showed a positive association with TBH at 10 years (β = 0.29, *p* = 0.011), indicating that initial bone availability contributed to long-term bone height stability. Prosthesis type, implant dimensions, gingival index, and smoking status were not significant predictors of TBH (*p* > 0.05).

## Discussion

The present retrospective trial aimed to assess the peri-implant tissue health and PROMs in indirect sinus floor elevation procedures with augmentation in patients with and without T2DM. A total of 76 patients with a mean age of 46.78 ± 11.96 years indicated for implant therapy in the posterior maxilla participated in this trial out of which 31 had well controlled T2DM while 45 belonged to non-T2DM group. The ten years follow-up was completed by 71 patients and five dropped out after four years of evaluation.

Prospective controlled trials are typically conducted under highly standardized and ideal conditions, where patient selection, operator variability, and treatment protocols are tightly regulated. While such designs minimize bias, they may not fully represent the complexity and diversity encountered in routine clinical practice. In contrast, observational retrospective studies capture real-world situations, including variations in patient compliance, systemic conditions, anatomical limitations, and clinician-dependent factors. These elements more closely mirror everyday practice, where patients often present with non-uniform characteristics and multifactorial influences on healing and treatment outcomes. Therefore, in the context of implant therapy—especially procedures such as indirect sinus floor elevation—a retrospective study may offer a more realistic understanding of long-term clinical performance and the practical success of the treatment modality across a broader patient population.

The prevalence of Diabetes has been increasing the world over and the metabolic control has been implicated as a modifying risk factor inflammatory conditions including peri implant diseases. Clinical parameters such as PD and radiographic bone loss, were found to be significantly higher in uncontrolled diabetic patients (HBA1c ≥ 8%), as compared to well-controlled diabetic patients (HBA1c < 8%) [20]. Gomez-Moreno et al. in another study it was found that higher HBA1c levels were associated with greater bone loss over 3 years after implant placement, though the results were not statistically significant [21]. Contrastingly no evidence of elevated HbA1c levels being associated with an increased marginal bone loss were exhibited in one of the prospective trials by Aguilar-Salvatierra et al. [22].

In the present study though the patients were diagnosed with well controlled T2DM the findings reveal that the parameters of MBL, SMT and PSTD were significantly greater than the non-T2DM group at the completion of two years post loading of implants. Similarly, there was a reduction in the mean TBH values in the T2DM group which indicated that the healing within the tissues was affected to a certain extent. These observations were also correspondingly exhibited on completion of four years and thereafter though the difference in mean values amongst the T2DM and non-T2DM groups of the overall parameters had less variations. On comparison of the baseline values with those at ten years follow-up in both the groups, MBL, SMT and PSTD were found to be greater while the TBH was significantly less. The modest annual reduction in TBH observed in both groups is consistent with the physiological remodeling that occurs within augmented sinus sites. After sinus floor elevation, graft biomaterials undergo gradual resorption and substitution by newly formed bone, which may result in minor dimensional changes over time depending on the resorption profile of the graft components used [23]. Additionally, coronal remodeling related to establishment of the peri-implant biologic width, microstrain at the implant–bone interface, and functional loading may contribute to slight crestal height modification [24]. Long-term follow-up studies have similarly demonstrated that such reductions represent normal maturation of the grafted sinus rather than pathological bone loss [25]. Importantly, these physiological changes did not compromise implant stability or survival in either metabolic group, underscoring the predictable long-term performance of the indirect sinus elevation approach.

The reason for TBH being less is the fact that it depends coronally on the crestal bone resorption while apically on the resorption of the graft particles. Also, though the parameters gained statistically significant differences, however clinically these did not bear a negative influence over the tangible benefits of implant therapy in such patients. These findings corroborate with the observations made in one of the systematic reviews and meta-analysis evaluating 252 patients and 587 dental implants wherein the authors found no significant association between diabetes and implant failure in patients with varying metabolic control [26]. The pooled risk ratio (RR) for overall implant failure was 0.620 (95% CI: 0.225–1.705), indicating no meaningful difference between well-controlled and poorly controlled diabetes.

Over the 10-year observation period, the overall implant survival rate was high in both groups, with 98.0% survival in the well-controlled T2DM group and 100% survival in the non-T2DM group. No implant mobility, persistent pain, or radiographic signs of loss of osseointegration were detected in surviving implants. Biological complications were limited and occurred more frequently in the T2DM cohort, primarily presenting as mild peri-implant mucositis, increased gingival index scores, and a higher frequency of peri-implant soft-tissue dehiscence. No cases of peri-implantitis meeting established diagnostic thresholds were observed in either group. Technical complications were minimal and included occasional screw loosening (T2DM: 2 cases; non-T2DM: 1 case), all of which were successfully managed without affecting implant function. No prosthesis fractures or major prosthetic failures occurred. These findings collectively indicate stable long-term implant performance in both metabolic groups, with slightly higher but manageable biological complications in the T2DM group.

Comparison of various PROMs between T2DM and non-T2DM groups from baseline to ten years follow-up, a significant difference of mean functional total score was observed while others exhibited non-significant differences. Since there were a few differences observed between the MBL and PSTD, there is bound to be some amount of hard and soft tissue deficiency. Such type of deficiencies leads to increased plaque formation which at times causes discomfort for the patient and is duly reflected in the findings of the present study. Since the diabetic status in the included patients was well controlled and the recall visits were strictly adhered to so there was minimal influence of the systemic status on the peri implant tissues in T2DM group.

One of the interesting aspects of the study was the use of composite graft with the intention of harnessing both the properties leading to a synergy of osteo-induction as well as osteo-conduction. Additionally, PRF containing concentrated platelets and growth factors releases them over a period of time generating a sustained regenerative ability during the healing phase. It is desired that the graft material used for augmentation create a conducive environment for invasion of blood vessels and bone forming cells thus inducing bone regeneration which also facilitates osseointegration of the inserted implants. It is also postulated that if the RBH is greater than 5 mm the primary stability required for the implant is provided by it and this enables undisturbed healing within the graft material which is extremely essential for the regeneration of tissues. During the bone healing period, insufficient initial stability may increase micromotion at the bone-implant surface, jeopardising the osseointegration process and thereby inducing fibrous tissue encapsulation [27]. The inclusion of patients with an RBH of 4 to 8 mm in the present study ensured that the healing goes uninterrupted.

Since the TBH achieved at the end of ten years in both the study groups was substantial as compared to baseline it can be affirmed that the graft not only led to new bone formation but also it was maintained on a longitudinal basis with appropriate osseointegration assessed from the implant stability. These findings are similar to those obtained by Wang et al. [28] in a clinical trial in which the vertical bone gain was correlated with the use of graft material with the observation period being three years. Above observations emphasize the effect of RBH and grafting material on new bone formation after lateral sinus augmentation using different biomaterials.

The results of the study indicate promising options for restoration of the masticatory apparatus in the posterior maxilla especially when the clinicians and patients are more inclined towards a minimal trauma and handling of the tissues, which is one of the major advantages of indirect sinus floor elevation with bone augmentation procedures.

The inclusion of multivariate regression analysis further strengthened the validity of the present findings by accounting for other variables that may influence peri-implant bone dynamics. After adjusting for oral hygiene status, prosthesis type, implant dimensions, smoking, and baseline RBH, diabetic status remained an independent predictor of increased MBL and reduced TBH at 10 years. This suggests that the metabolic alterations associated with T2DM may have a unique and measurable effect on peri-implant bone remodeling, beyond the influence of local mechanical or prosthetic factors. These results also highlight that while oral hygiene and prosthetic design are important for peri-implant health, they did not independently affect long-term bone stability in this cohort.

The retrospective design did not allow control over past periodontal history and light smoking status, which may be one of the limitations of the study. Nevertheless, the results of this retrospective study should be interpreted with caution due to a smaller sample size, operator and assessor variability and inability to determine the bone density. PROMs is a subjective variable which cannot determine actual correlation between the overall clinical results and patient satisfaction.

## Conclusion

Within the limitations of this retrospective study, indirect sinus floor elevation with composite graft demonstrated predictable and successful long-term outcomes in both well-controlled T2DM and non-T2DM patients. Although both groups showed stable implants and satisfactory function over the 10-year period, non-T2DM patients exhibited greater bone regeneration, reflected by significantly higher TBH and SMT values. The T2DM group showed higher MBL and greater PSTD values, suggesting a modest compromise in tissue healing despite overall treatment success. Functional scores also favored the non-T2DM group. These findings indicate that well-controlled T2DM does not preclude favorable long-term outcomes following indirect sinus augmentation, although subtle differences in bone and soft tissue responses persist. Future prospective studies with larger cohorts and standardized metabolic monitoring are warranted to further clarify the influence of glycemic control on graft maturation, soft-tissue stability, and functional implant performance.

## Data Availability

The datasets generated and/or analysed during the current study are available from the corresponding author on request.
